# Reviewer acknowledgement

**DOI:** 10.1186/s40560-015-0074-7

**Published:** 2015-03-12

**Authors:** Satoshi Gando

**Affiliations:** Hokkaido University Graduate School of Medicine, ᅟ, Hokkaido Japan

## Abstract

*Journal of Intensive Care* is very grateful for the generously-provided time and constructive, insightful reports provided by the journal’s highly-qualified reviewers. We would like to show our appreciation by thanking the following people for their assistance with review of manuscripts for the journal in 2014.

**KunTae Ahn**

Republic of Korea

**Mayuki Aibiki**

Japan

**Fumimasa Amaya**

Japan

**Hideki Arimoto**

Japan

**Kevin Clarkson**

Ireland

**Colin Cookee**

United States of America

**Jigeeshu Divatia**

India

**Moritoki Egi**

Japan

**Yutaka Eguchi**

Japan

**Takashi Etoh**

Japan

**Raphael Favory**

France

**Seitaro Fujishima**

Japan

**Yoshihito Fujita**

Japan

**Satoshi Fujita**

Japan

**Shigeki Fujitani**

Japan

**Tatsuma Fukuda**

Japan

**Hiraku Funakoshi**

Japan

**Harumi Gomi**

Japan

**Satoru Hanada**

Japan

**Go Haraguchi**

Japan

**Satoru Hashimoto**

Japan

**Mineji Hayakawa**

Japan

**Yoshiro Hayashi**

Australia

**Patrick Honore**

Belgium

**Toshiaki Ikeda**

Japan

**Takanari Ikeyama**

Japan

**Hideaki Imanaka**

Japan

**Hideo Inaba**

Japan

**Satoki Inoue**

Japan

**Shigeaki Inoue**

Japan

**Yoshiaki Inoue**

Japan

**Hironori Ishihara**

Japan

**Hiroyasu Ishikura**

Japan

**Eiji Isotani**

Japan

**Takashi Ito**

Japan

**Motoshi Kainuma**

Japan

**Yasuyuki Kakihana**

Japan

**Shunji Kasaoka**

Japan

**Seiya Kato**

Japan

**Nobuyuki Katori**

Japan

**Atsushi Kawaguchi**

Canada

**Kaneyuki Kawamae**

Japan

**Tatsuya Kawasaki**

Japan

**Kosaku Kinoshita**

Japan

**Andoh Kohkichi**

Japan

**Yutaka Kondo**

Japan

**Yoshifumi Kotake**

Japan

**Joji Kotani**

Japan

**Toru Kotani**

Japan

**Junji Kumasawa**

Japan

**Yasuhiro Kuroda**

Japan

**Shigeki Kushimoto**

Japan

**Jeffrey Lipman**

Australia

**Graeme MacLaren**

Singapore

**Seiji Madoiwa**

Japan

**John Marshall**

Canada

**Kazuo Maruyama**

Japan

**Yoshiki Masuda**

Japan

**Hikoro Matsui**

Japan

**Hiroyuki Matsuoka**

Japan

**Francisco Maynar**

Spain

**Hiroyuki Mima**

Japan

**Chieko Mitaka**

Japan

**Hiroshi Morimatsu**

Japan

**Yuji Morimoto**

Japan

**Hiroshi Morisaki**

Japan

**Takashi Moriya**

Japan

**Kiyoshi Moriyama**

Japan

**Takaya Morooka**

Japan

**Satoshi Nakagawa**

Japan

**Masaki Nakane**

Japan

**Minoru Nakano**

Japan

**Atsunori Nakao**

Japan

**Hiromichi Narumiya**

Japan

**Shinichi Nishi**

Japan

**Hiroshi Nishida**

Japan

**Masaji Nishimura**

Japan

**Kenji Nishio**

Japan

**Akira Nishisaki**

United States of America

**Takeshi Nomura**

Japan

**Shin Nunomiya**

Japan

**Ryoichi Ochiai**

Japan

**Shigeto Oda**

Japan

**Kazufumi Okamoto**

Japan

**Desebbe Olivier**

France

**Mutsuo Onodera**

Japan

**Giles Peek**

United Kingdom

**Hiroshi Rinka**

Japan

**Yoshiro Sakaguchi**

Japan

**Sanju Samuel**

United States of America

**Nobuo Sasaki**

Japan

**Teiji Sawa**

Japan

**Naoki Sawa**

Japan

**Atsushi Sawamura**

Japan

**Hirotaka Sawano**

Japan

**Nobuaki Shime**

Japan

**Naoki Shimizu**

Japan

**Yoshito Shiraishi**

Japan

**Naosuke Sugai**

Japan

**Nakaba Sugimoto**

Japan

**Hidemichi Suyama**

Japan

**Takeshi Suzuki**

Japan

**Naoyuki Taga**

Japan

**Yoshio Tahara**

Japan

**Shinhiro Takeda**

Japan

**Tetsuhiro Takei**

Japan

**Naoshi Takeyama**

Japan

**Ryoma Tanaka**

United States of America

**Yoshiaki Terao**

Japan

**Kentaro Tokuda**

Japan

**Nai Shun Nelson Tsoi**

Hong Kong

**Ryosuke Tsuruta**

Japan

**Shigehiko Uchino**

Japan

**Masahiko Uzura**

Japan

**Jean Louis Vincent**

Belgium

**Hiroshi Yamada**

Japan

**Taihei Yamada**

Japan

**Takeshi Yamamoto**

Japan

**Akira Yamashina**

Japan

